# Microbiome Associated with Slovak Raw Goat Milk, Trace Minerals, and Vitamin E Content

**DOI:** 10.1155/2022/4595473

**Published:** 2022-08-30

**Authors:** Andrea Lauková, Lenka Micenková, Ľubomíra Grešáková, Michaela Maďarová, Monika Pogány Simonová, Valentína Focková, Jana Ščerbová

**Affiliations:** ^1^Centre of Biosciences of the Slovak Academy of Sciences, Institute of Animal Physiology, Šoltésovej 4-6, 040 01 Košice, Slovakia; ^2^Recetox, Faculty of Science, Masaryk University, Kotlářska 2, 611 37 Brno, Czech Republic

## Abstract

In Slovakia, goat milk production for direct consumption and cheese processing has attracted growing interest. However, there is a lack of information regarding the microbial consortium in Slovak raw goat milk analyzed by next-generation sequencing and trace elements and vitamin E as well. A randomly selected samples (G24-G50) of raw goat milk from different animals at farms in Slovakia were analyzed. The phylum Actinobacteria dominated (62.8%), followed by the phyla Firmicutes (20.5%), Proteobacteria (7.4%), and Bacteroidetes (6.4%). The family *Microbacteriaceae* was detected in the highest percentage (60.2%) followed by *Staphylococcaceae*, *Bacteroidaceae*, *Streptococcaceae*, *Lactobacillaceae*, *Enterobacteriaceae*, and others. Regarding the genera, the most prevalent was genus *Curtobacterium* (47.4%) followed by the genera such as *Staphylococcus* (8.3%) and *Bifidobacterium* (4%). The genera *Streptococcus*, *Lactococcus*, *Enterococcus*, *Lactobacillus*, and *Lacticaseibacillus* were evaluated in abundance percentage in range 1%-3.2%. The genus *Veillonella* reached abundance 3.2%. The genera *Enterobacter*, *Pseudomonas* (1.3% and 0.5%), and *Bacteroides* (6.4%) were evaluated in small percentage abundance too. Zinc was detected with the highest mean value (2.561 ± 0.6823 mg/L) in raw goat milk, followed by iron (1.383 ± 0.5087 mg/L). The mean value of copper and manganese was 0.1746 ± 0.0463 mg/L and 0.051 ± 0.0238 mg/L. The vitamin E reached the mean value 0.3783 ± 0.1976 mg/L. This study is an original contribution showing microbial consortium in raw goat milk from Slovak farms. It also contributes to trace elements and vitamin E status in raw goat milk showing it as a nutritionally healthy food.

## 1. Introduction

The breeding of goats has had a long tradition in Slovakia, especially on small farms. Moreover, goat breeding is often associated with agrotourism. Nowadays, goat milk production for direct consumption and cheese processing has attracted growing interest [1]. Goat milk is a highly nutritive product that has advantages over other milk. The immunoglobulin is more abundant (up to 80 *μ*g/mL) than for example in breast milk [2]. In addition, the contents of protein (3.43%), fat (3.21%), minerals (up to 150 mg in case of Cl), vitamins (vitamin E, 0.4-0.11 mg/kg), and other nutrients of goat milk are much higher than other milk [3, 4]. Goat milk is, for example, more valuable in iron (Fe, 0.07-1.025 mg/L), copper (Cu, 0.05 mg and higher), zinc (Zn, 0.56 mg), and manganese (Mn, 0.032 mg) than, e.g., cow milk [5, 6]. However, mineral content can vary during the lactation period, which closely relates to the production season [7]. The content of vitamin E usually increases during the lactation period, e.g., up to 0.11 mg/kg [7]. Besides many general benefits, raw milk is also a beneficial medium for the growth of food-borne pathogens and spoilage psychrotrophic bacteria [8]. Although strict hygienic and zoohygienic conditions are kept during milk processing, spoilage microbiota, e.g., staphylococci, can contaminate goat milk because of farming production [9]. There exist studies mapping the occurrence of beneficial and/or spoilage bacteria in raw milks using standard microbiological analyses [9, 10, 11, 12, 13, 14] and also next-generation sequencing techniques [10, 14, 15]. However, regarding the microbial consortium (microbiome) mapping in Slovak raw goat milk by using next-generation sequencing technique only, a lack of information exists. To know microbiome composition allows later preventing/avoiding of contaminant bacteria. At the same time, it also allows to screen beneficial bacterial phyla or genera occurrence for their representative selection. Therefore, to bring an original contribution in dairy microbiology, the principal aim of this study was to assess the microbiome of Slovak raw goat milk sampled from different Slovak farms. For this purpose, next-generation sequencing technique was used. Additional aim was to evaluate the amount of vitamin E and trace elements in goat milk because of limited data in Slovakia.

## 2. Material and Methods

### 2.1. Milk Sampling and Microbiome Analysis

A total of 53 raw goat milk samples from healthy goats were collected in central and eastern Slovakia farms involving 283 goats as previously reported by Lauková et al. [13, 14]. Sampling was performed from 51 individual goats, and two pooled milk samples were taken [13, 14]. Those milk samples were treated using the standard microbiological method as previously mentioned by Lauková et al. [13, 6, 9]. However later, for next-generation technique analysis, lastly sampled 26 milks (G24-G50) from different animals were used. Animals of the breed white shorthaired goat from two farms were sampled during late spring and summer period (morning milking). Goats were on pasture (outdoor feeding). After milk transport to the laboratory in a refrigerating box, the appropriate volume of raw goat milk samples (100 mL) was frozen until further analyses. A 260 *μ*L of raw goat milk was used for the DNA isolation performed by DNeasy PowerLyzer PowerSoil Kit (QIAGEN, Hilden, Germany) according to the manufacturer's protocol. Isolated DNA was used as a template in PCR reaction targeting the hypervariable V4 region of the bacterial 16S rRNA gene according to the 16S Metagenomic Sequencing Library Preparation protocol (Illumina, San Diego, CA, USA). The PCR detection protocol was as follows: 98°C (30 seconds), 98°C (10 seconds), 55°C (15 seconds), 72°C (25 seconds), and 30 cycles 72°C (2 min) as also reported in our previous study [15]. The primer pairs used are listed in Table 1. Sequencing was performed using MiSeq reagents Kits v2 on a MiSeq 2000 sequencer according to the manufacturers' instructions (Illumina, San Diego, CA, USA).

### 2.2. Mineral Analysis

Trace mineral concentrations (Zn, Fe, and Cu) in raw goat milk samples were determined by flame atomic absorption spectrometry (FAAS) in an air-acetylene flame, with deuterium background correction, using an atomic absorption spectrophotometer AA-7000 (Shimadzu Co., Kyoto, Japan) with a graphite furnace (GFA-7000, Shimadzu Co., Kyoto, Japan). Manganese (Mn) concentration in milk was determined using a graphite furnace atomic absorption spectrophotometer with deuterium background correction and pyrolytic-coated graphite tubes. The microwave-assisted digestion method using closed pressure vessels in an MWS4 Speedwave device (Berghof Co., Eningen, Germany) was used for the decomposition of milk samples in two replicates. The certificate reference material of skimmed milk powder (ERM-BD151) from the Institute for Reference Materials and Measurements-IRMM (Geel, Belgium) was included in each analysis to verify the instrument's accuracy. Mineral milk concentration was expressed in mg/L [16]. Working standard solution of trace elements from 1000 mg/L certified stock solutions (Ultra Scientific, North Kingstown, RI, USA) was used, and analytical calibration standards were prepared by dilution in 2% HNO_3_. All reagents were analytical reagent grade, and the ultrapure water used for solutions was purified with an ultrapure water system (EASYpure II UV/UF, Werner Reinstwassersysteme, Leverkusen, Germany). All plastic vials, tubes, autosampler cups, tips, and glassware materials were cleaned, soaked in 20% HNO_3_ for 24 hours, rinsed three times with ultrapure water, and dried before use in the sample collection and analyses.

### 2.3. Vitamin E (Tocopherol) Content Analysis

The concentration of vitamin E was analyzed using a high-performance liquid chromatography (HPLC, UltiMate 3000, Dionex, Germany). Vitamin E was separated on a Phenomenex Jupiter 5u C18 300A column (250 × 4.6 mm, 5 *μ*m). Methanol (100%) was used as the mobile phase at a 1 mL/min flow rate. DAD detection of *α*-tocopherol was performed at 292 nm in a sample injection of 20 *μ*L at room temperature. Vitamin E was eluted at a retention time of 6.6 min. Concentration of vitamin E in milk was expressed in mg/L. Each milk sample was measured three times. Samples of goat milk were deproteinized with methanol and then extracted with n-hexane (Merck, Germany). The extract was vortexed and centrifuged at 10,000 × *g* for 8 min (Jouan MR 1812, Saint Herblain, France). The organic phase was collected and evaporated to dryness under nitrogen. The residue was dissolved in 100% methanol. The samples were injected into a chromatographic column (Phenomenex Jupiter, 5u C18 300A column (250 × 4.6 mm, 5 *μ*m), and analytes were detected using an external standard calibration (Laboratory of Genetic Ecotoxicology in Kolín, Institute of Experimental Medicine, Academy of Sciences, Czech Republic, Prague).

### 2.4. Data Analysis

Statistical analyses were performed by the GraphPad Prism 8 statistical software (GraphPad Prism version 5.0, GraphPad Software, San Diego, CA, USA). Regarding the mineral, measured values were processed by analysis of variance (ANOVA), using the post hoc Tukey's test. Data are expressed as mean and standard deviation (SD). In vitamin E analysis, the linearity of the calibration curve for *α*-tocopherol was obtained at six concentrations ranging from 0.05 to 30.0 mg/L based on peak area (*r* = 0.99993). Data were summarized using chromatographic software (Chromeleon Chromatography Management System Version 6.80, Dionex 2006, Germany).

**Figure 1:**
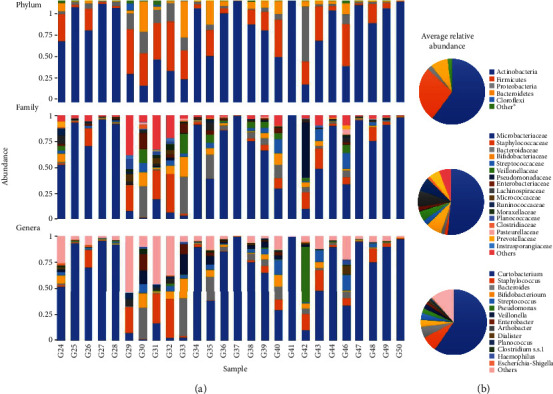
Relative abundance of the bacterial population analyzed from the sequencing of the 16S rRNA gene in DNA samples isolated from goat milk at the phylum, family, and genus level. (a) Bar chart of relative abundances of the bacteria across each sampled goat milk (*n* = 26). (b) Pie chart of the average relative abundances of the bacteria. ^∗^Bacteria with relative abundance higher than 0.5% (at phylum level) and 10% (at the family and genera levels) are visualized (taxa lower than 1% are displayed as “others”).

Regarding Figure 1, relative abundance of the bacterial population is analyzed from the sequencing of the 16S rRNA gene in DNA samples isolated from goat milk at the phylum, family, and genus level.  Figure 1(a) shows the bar chart of relative abundances of the bacteria across each sampled goat milk (*n* = 26).  Figure 1(b) shows the pie chart of the average relative abundances of the bacteria. Bacteria with relative abundance higher than 0.5% (at phylum level) and 10% (at the family and genera levels) are visualized (taxa lower than 1% are displayed as “others”).

## 3. Results

### 3.1. Microbiome Detected in Slovak Raw Goat Milk

The phylum Actinobacteria dominated in raw goat milk samples (62.8%), followed by the phyla Firmicutes (20.5%), Proteobacteria (7.4%), and Bacteroidetes (6.4%) (Figure 1). The family *Microbacteriaceae* was found in the highest abundance percentage (60.2%). The genus *Curtobacterium* (47.4%) (Figure 1) was the most prevalent in Slovak raw goat milk. It belongs to the family *Microbacteriaceae* and to the phylum Actinobacteria. The second genus detected in the framework of the phylum Actinobacteria was the genus *Bifidobacterium* belonging to the family *Bifidobacteriaceae*. It was detected with low percentage abundance (3.5%); abundance achieved for the genus *Bifidobacterium* was almost the same (4%) (Figure 1). The phylum Firmicutes found with an abundance of 20.4% in analyzed Slovak raw goat milk was represented by Gram-positive genera such as *Staphylococcus*, *Streptococcus*, *Lactococcus*, *Lactobacillus*, *Lacticaseibacillus*, and *Enterococcus* and by one Gram-negative genus *Veillonella* (Figure 1). The genus *Staphylococcus* was the most prevalent (8.3%), followed by the genera *Streptococcus* (3.2%), *Lactococcus*, *Enterococcus*, *Lactobacillus*, and *Lacticaseibacillus* detected in low abundance (up to 1%, Figure 1). Genera detection was in association with the appropriate families detected; *Staphylococcaceae* reached abundance 7.2%, followed by *Streptococcaceae* (3.5%) as well as families (*Lactococcaceae*, *Lactobacillaceae*, and *Enterococcaceae*). The Gram-negative genus *Veillonella* with abundance 3.2% belongs in the family *Veillonellaceae* (abundance 2.73%). Proteobacteria were represented by the genera *Enterobacter*, *Pseudomonas*, and *Moraxella* (1.3% and 0.5%). The genus *Pseudomonas* belongs in the family *Pseudomonadaceae*, an abundance of which reached up to 1%. The genus *Moraxella* from the family *Moraxellaceae* (1.3%) is also part of Proteobacteria, and the phylum Bacteroidetes was represented by the genus *Bacteroides* (6.4%) from the family *Bacteroidaceae* (5.5%); the family *Prevotellaceae* was also detected (up to 1%). In the family *Enterobacteriaceae* (2.4% abundance) framework, the genus *Enterobacter* (1.2%) was determined. Besides the family *Microbacteriaceae a*nd formerly mentioned, the other families were determined in very low abundances such as *Pasteurellaceae*, *Lachnospiraceae*, *Planococcaceae*, *Ruminococcaceae*, *Aerococcaceae*, *Clostridiaceae*, and/or *Intrasporangiacae*. Also the genera Arthrobacter, *Dialister*, *Planococcus*, *Clostridium SS1*, *Escherichia-Shigella*, or *Haemophilus* were determined (Figure 1).

### 3.2. Trace Elements and Vitamin E (Tocopherol) Contents in Raw Goat Milk

Results regarding the minerals and vitamin E analyses are summarized in Table 2. The highest mean value in raw goat milk was reached for Zn (2.561 ± 0.6823 mg/L). Iron (Fe) concentration was also high (1.383 ± 0.5087 mg/L). The mean value of measured Cu was 0.1746 ± 0.0463 mg/L. Manganese was evaluated in the lowest concentration in raw goat milk analyzed (0.051 ± 0.0238 mg). The mean value of vitamin E concentration reached 0.3783 ± 0.1976 mg/L).

## 4. Discussion

To our knowledge, it is the first study using the next-generation sequencing technique to detect different microbial taxa in Slovak raw goat milk. Based on the results, the phylum Actinobacteria dominated, followed by the phyla Firmicutes, Proteobacteria, and Bacteroidetes. Parente et al. [12] reported the most prevalent and abundant taxa in raw cow milk and teat cow milk belonging to the phyla Firmicutes, Actinobacteria, and Bacteroidetes. Metzger et al. [17] mentioned that seasonal effect is noted not only for trace elements and vitamin E content in milk but also for microbiota in milk. The phylum Firmicutes found with abundance 20.4% in analyzed raw goat milk was represented by the genera such as *Staphylococcus*, *Streptococcus*, *Lactococcus*, *Lactobacillus*, *Lacticaseibacillus*, *Enterococcus*, and *Veillonella.* Also the genera *Arthrobacter*, *Dialister*, *Planococcus*, *Clostridium SS1*, *Escherichia-Shigella*, or *Haemophilus* were determined, however with very low abundance. The prevalence of the genus *Staphylococcus* was found; it is in association with the most frequently detected staphylococcal species in raw goat milk as previously reported by Lauková et al. [13]. The following 14 species were detected, which can be involved in seven different clusters (*Staphylococcus arlettae*, *S. capitis*, *S. delphini*, *S. epidermidis*, *S. equorum*, *S. hominis*, *S. lentus*, *S. pasteuri*, *S. sciuri*, *S. simulans*, *S. schleiferi*, *S. vitulinus*, *S. warneri*, and *S. xylosus*. The highest staphylococcal occurrence in raw goat milk can be associated with teat handling [12]. Also potential mastitis agents can be abundant components of the milk microbiota [17, 18]. However, potentially beneficial bacterial genera were also detected in raw goat milk (*Lacticaseibacillus*, *Lactococcus*, *Enterococcus*, and *Streptococcus*). Although the representatives of the genera including mainly lactic acid bacteria were detected in slight amount percentage in raw goat milk, they can play a key role in microbiota ratio optimizing in goat milk due the production of not only lactic acid but also bacteriocins [19, 20, 6]. Bacteriocins produced mainly by some enterococci [19, 20, 21, 6] can be used to reduce/protect goat milk against spoilage bacteria such as those from detected phyla Bacteroidetes or Proteobacteria and families *Pseudomonadaceae*, *Moraxellaceae*, *Enterobacteriaceae*, and/or *Clostridiaceae*. The antimicrobial effect of enterocins against representatives of the families mentioned was already reported [22, 23, 13]. However, the antimicrobial effect can interact also with lactic acid production. On the other hand, some representative strains of LAB from the phylum Firmicutes can be studied for their beneficial properties and used as a dairy additive.

Besides standard microbiological methods used to identify microbiota in raw goat milk, an advantage of next-generation technique is possibility to detect microbial consortia present only in limited amounts in milk or that exist in a viable but nonculturable state [24]. Therefore, for knowing milk composition, this method is useful as shown also in this study. Kamilari et al. [25] indicated the genera *Bacteroides*, *Staphylococcus*, and *Streptococcus* but also *Pseudomonas*, *Acinetobacter*, or *Burkholderia* in different goat milks (from different regions) and stored at different temperatures. They mentioned that raw milk storage conditions may affect the microbial composition. Storage at 4°C may lead to the development of psychrotrophic bacteria. Our sampling was performed mainly during milking late spring and summer period which could also impact milk composition. In our case, animals were in outdoor feeding.

As formerly mentioned, goat milk is a more valuable source of Fe, Cu, Zn, and Mn than cow milk [26, 27, 5]. However, the chemical composition of milk, including the microelements content, depends on a variety of environmental, genetic, and physiological factors [28]. Barlowska et al. [5] and Michlová et al. [7] reported seasonal effects on mineral element content in goat milk. Similarly, Pavlata et al. [29] and Rolinec et al. [30] reported the effect of season and lactation stage effects on Cu and Zn concentrations in goat milk. They analyzed the highest concentration of Cu, Fe, and Zn in goat milk derived from goats which were seven days on pasture. Those mineral amounts were higher than those detected in goat milk derived from indoor feeding goats. The start of grazing of dairy goats affects the nutritive and mineral composition of goat milk [30].

In general, the contents of zinc, copper, and iron have a decreased tendency throughout lactation, while reverse tendency was noted regarding the Mn content in milk [31]. Coni et al. [32] reported that goat milk sampled in the summer had a higher content of Cu, Fe, and manganese, while winter milk had higher levels of Mn and Zn. Kondyli et al. [33] also observed seasonal changes in the most important macroelements, i.e., calcium, phosphorus, and potassium, and microelements, i.e., Cu and Zn. Milk sampled from goats in the I stage of lactation (winter feeding) was the richest source of Zn, Fe, and Cu, and milk from III stage (autumn-winter feeding) was the highest in Mn. Concentrations of trace minerals are affected by diet, breed, animal, and stages of lactation [34]. The average mineral content in goat milk is higher than that of cow milk. However, goat milk has a lower degree of hydration and has an inverse relationship between the mineralization of the micelle and its hydration [35].

Michlová et al. [36] reported the vitamin E content increase almost continuously during the lactation. The average content of vitamin E, e.g., in Saanen goat milk, was higher (2.2 times) than, e.g., the content of vitamin A [36, 7]. A higher level of vitamin E in goat milk is also reported by Morand-Fehr et al. [37] or Park et al. [4]. There was reported a high value of vitamin E (up to 11 mg/kg) depending on the farming and feeding system. On the contrary, a lower value of vitamin E (0.4 mg/kg) in goat milk was reported by Reynal-Ljutovac et al. [38]. Nowadays, the term functional food is very frequently used in health concept. However, more definitions exist. One definition claimed the functional food as a food with additional function or new ingredients, beneficially influencing physiological status. Another definition presents the functional food to have beneficial effect on health beyond basic nutrition, and it supports optimal health and can lower disease. In the other definition, the functional food is the gradual recognition that healthy diets result to eating nutritious food and identification of mechanism by which food modulates metabolism and health. And also combination of some definitions such as functional food is that one if it is satisfactorily demonstrated to affect beneficially one or more target functions in the body, beyond adequate nutritional effects, in a way that is relevant to either improved state of health and well-being and/or reduction of disease risk (EC Project FUFOSE Consensus Document 1999). But the simplest term is the functional food = nutritionally healthy food. Therefore, knowing microbial status, vitamins, and mineral content indicates raw goat milk as functional food in connection with various definitions.

## 5. Conclusion

Microbiome of Slovak raw goat milk was analyzed at different levels. The phylum Actinobacteria dominated, followed by the phyla Firmicutes, Proteobacteria, and Bacteroidetes. At the family level, *Microbacteriaceae* dominated, followed mainly by the families *Staphylococcaceae*, *Bacteroidaceae*, *Streptococcaceae*, and *Veillonellacea*e. The genera detected with higher abundance percentage were *Curtobacterium*, *Staphylococcus*, *Bifidobacterium*, *Streptococcus*, *Lactococcus*, *Enterococcus*, *Lactobacillus*, *Lacticaseibacillus*, *Veillonella*, *Bacteroides*, *Enterobacter*, and *Pseudomonas*. Among minerals, zinc reached the highest mean value, followed by the iron. Cu and Mn contents were lower. The mean value of vitamin E reached up to 0.3783 mg/L. The microbiome of Slovak raw goat milk has not been analyzed at these levels. Therefore, this study is an original contribution in how to show a key role of microbiota in goat milk to maintain a quality of milk. Altogether, assessing microbiota, mineral, and vitamin content in raw goat milk indicates the possibility to show goat milk as nutritionally healthy functional food.

## Figures and Tables

**Table 1:** List of primers.

EMP16S-1	F	TCGTCGGCAGCGTCAGATGTGTATAAGAGACAGAGCCTTCGTCGCGTGTGYCAGCMGCCGCGGTAA	16S metagenomic sequencing library preparation protocol; Illumina, USA (EMP 515-806)
	R	GTCTCGTGGGCTCGGAGATGTGTATAAGAGACAGCCTAACGGTCCACCGGACTACNVGGGTWTCTAAT	
EMP16S-2	F	TCGTCGGCAGCGTCAGATGTGTATAAGAGACAGTCCATACCGGAAGTGTGYCAGCMGCCGCGGTAA	
	R	GTCTCGTGGGCTCGGAGATGTGTATAAGAGACAGCGCGCCTTAAACCCGGACTACNVGGGTWTCTAAT	
EMP16S-3	F	TCGTCGGCAGCGTCAGATGTGTATAAGAGACAGAGCCCTGCTACAGTGTGYCAGCMGCCGCGGTAA	
	R	GTCTCGTGGGCTCGGAGATGTGTATAAGAGACAGTATGGTACCCAGCCGGACTACNVGGGTWTCTAAT	
EMP16S-4	F	TCGTCGGCAGCGTCAGATGTGTATAAGAGACAGTGAGACCCTACAGTGTGYCAGCMGCCGCGGTAA	
	R	GTCTCGTGGGCTCGGAGATGTGTATAAGAGACAGGCCTCTACGTCGCCGGACTACNVGGGTWTCTAAT	
EMP16S-5	F	TCGTCGGCAGCGTCAGATGTGTATAAGAGACAGACTTGGTGTAAGGTGTGYCAGCMGCCGCGGTAA	
	R	GTCTCGTGGGCTCGGAGATGTGTATAAGAGACAGACTACTGAGGATCCGGACTACNVGGGTWTCTAAT	
EMP16S-6	F	TCGTCGGCAGCGTCAGATGTGTATAAGAGACAGATTACGTATCATGTGTGYCAGCMGCCGCGGTAA	
	R	GTCTCGTGGGCTCGGAGATGTGTATAAGAGACAGAATTCACCTCCTCCGGACTACNVGGGTWTCTAAT	
EMP16S-7	F	TCGTCGGCAGCGTCAGATGTGTATAAGAGACAGCACGCAGTCTACGTGTGYCAGCMGCCGCGGTAA	
	R	GTCTCGTGGGCTCGGAGATGTGTATAAGAGACAGCGTATAAATGCGCCGGACTACNVGGGTWTCTAAT	
EMP16S-8	F	TCGTCGGCAGCGTCAGATGTGTATAAGAGACAGTGTGCACGCCATGTGTGYCAGCMGCCGCGGTAA	
	R	GTCTCGTGGGCTCGGAGATGTGTATAAGAGACAGATGCTGCAACACCCGGACTACNVGGGTWTCTAAT	
EMP16S-9	F	TCGTCGGCAGCGTCAGATGTGTATAAGAGACAGCCGGACAAGAAGGTGTGYCAGCMGCCGCGGTAA	
	R	GTCTCGTGGGCTCGGAGATGTGTATAAGAGACAGACTCGCTCGCTGCCGGACTACNVGGGTWTCTAAT	
EMP16S-10	F	TCGTCGGCAGCGTCAGATGTGTATAAGAGACAGTTGCTGGACGCTGTGTGYCAGCMGCCGCGGTAA	
	R	GTCTCGTGGGCTCGGAGATGTGTATAAGAGACAGTTCCTTAGTAGTCCGGACTACNVGGGTWTCTAAT	
EMP16S-11	F	TCGTCGGCAGCGTCAGATGTGTATAAGAGACAGTACTAACGCGGTGTGTGYCAGCMGCCGCGGTAA	
	R	GTCTCGTGGGCTCGGAGATGTGTATAAGAGACAGCGTCCGTATGAACCGGACTACNVGGGTWTCTAAT	
EMP16S-12	F	TCGTCGGCAGCGTCAGATGTGTATAAGAGACAGGCGATCACACCTGTGTGYCAGCMGCCGCGGTAA	
	R	GTCTCGTGGGCTCGGAGATGTGTATAAGAGACAGACGTGAGGAACGCCGGACTACNVGGGTWTCTAAT	
EMP16S-13	F	TCGTCGGCAGCGTCAGATGTGTATAAGAGACAGCAAACGCACTAAGTGTGYCAGCMGCCGCGGTAA	
	R	GTCTCGTGGGCTCGGAGATGTGTATAAGAGACAGGGTTGCCCTGTACCGGACTACNVGGGTWTCTAAT	
EMP16S-14	F	TCGTCGGCAGCGTCAGATGTGTATAAGAGACAGGAAGAGGGTTGAGTGTGYCAGCMGCCGCGGTAA	
	R	GTCTCGTGGGCTCGGAGATGTGTATAAGAGACAGCATATAGCCCGACCGGACTACNVGGGTWTCTAAT	
EMP16S-15	F	TCGTCGGCAGCGTCAGATGTGTATAAGAGACAGTGAGTGGTCTGTGTGTGYCAGCMGCCGCGGTAA	
	R	GTCTCGTGGGCTCGGAGATGTGTATAAGAGACAGGCCTATGAGATCCCGGACTACNVGGGTWTCTAAT	

**Table 2:** Trace mineral profile and vitamin E amount in Slovak raw goat milk.

	Mean	SD	Minimum	Maximum
Mineral concentration (mg/L) (*n* = 26)				
Zinc	2.561	0.6823	1.421	3.83
Iron	1.383	0.5087	0.63	2.49
Copper	0.1746	0.0463	0.0845	0.249
Manganese	0.051	0.0238	0.018	0.092
Vitamin concentration (mg/L)				
Vitamin E (tocopherol)	0.3783	0.1976	0.1439	0.8313

Mean value ± SD.

## Data Availability

The data that support the findings of this study are available on request from the corresponding author (A.L).
